# Improving Type 2 Diabetes Prediction: Comparative Evaluation of Machine Learning Classifiers Using Balanced Data from the AWI-Gen Cohort

**DOI:** 10.21203/rs.3.rs-8019155/v1

**Published:** 2025-11-04

**Authors:** Richmond Balinia Adda

**Affiliations:** 1 Department of Biometry, School of Mathematical Science, C. K. Tedam University of Technology and Applied Science (UTAS), Navrongo, Ghana; 2 Navrongo Health Research Centre, Ghana Health Service, Navrongo, Ghana; 3 Department of Biostatistics & Epidemiology, School of Public Health, C. K. Tedam University of Technology and Applied Science (UTAS), Navrongo, Ghana

**Keywords:** Machine Learning, Type 2 Diabetes, Digital Health, Predictive Modelling, Africa, Clinical Validation, XGBoost

## Abstract

**Background:**

Type 2 diabetes mellitus (T2DM) is an escalating public health concern across Africa, but regionally tailored predictive models are scarce. Advances in machine learning (ML) offer potential for early identification, though previous research has been constrained by methodological issues such as data leakage, class imbalance, and overfitting, limiting clinical deployment, especially in digital health contexts.

**Methods:**

This study analysed data from 2,010 participants in the H3Africa AWI-Gen cohort in northern Ghana to develop and evaluate ML-based prediction models tailored to African settings. Rigorous preprocessing steps, including handling class imbalance with SMOTE and excluding diagnostic biomarkers prone to target leakage, were applied. Eight ML classifiers underwent robust Bayesian hyperparameter optimisation. Model performance was assessed via stratified 5-fold cross-validation and confirmed through extensive sensitivity and calibration analyses.

**Results:**

The optimised XGBoost model yielded an AUC of 0.845 (95% CI: 0.812–0.878) and a sensitivity of 78.2% on unseen data. Including glucose as a predictor increased performance by 11.5%, underscoring the necessity of its exclusion to avoid biased evaluation. Models using only anthropometric and lifestyle variables (AUC = 0.783) demonstrated robust predictive capacity, with waist circumference, physical activity, and BMI standing out as the most stable predictors across analyses.

**Conclusion:**

Our findings demonstrate that ML models constructed from routinely collected clinical and lifestyle data can attain clinically meaningful diabetes prediction suitable for digital health applications in low-resource African contexts. This study addresses prior methodological gaps and offers a data-driven framework that is both robust and clinically plausible for early T2DM detection, with potential implications for public health policy and digital screening programmes in similar populations.

## 1 Introduction

Type 2 diabetes mellitus (T2DM) represents a mounting global health crisis, with particularly severe implications for African populations experiencing rapid epidemiological transitions. Current estimates indicate approximately 25 million adults in Africa live with diabetes, with projections suggesting an alarming 140

Early T2DM identification is critical for mitigating the substantial morbidity and mortality associated with delayed diagnosis, including debilitating microvascular and macrovascular complications that impose considerable strain on healthcare systems [12]. Traditional risk assessment predominantly relies on biochemical markers that remain impractical in many low- and middle-income countries (LMICs), creating substantial detection gaps that perpetuate health disparities.

Machine learning (ML) has emerged as a promising alternative for diabetes prediction by capturing complex, nonlinear interactions among diverse risk factors. Multiple studies have demonstrated the potential of various ML classifiers, including random forests, support vector machines, and gradient boosting algorithms for metabolic disease prediction [20, 7]. However, methodological limitations—particularly data leakage and overfitting—have frequently led to artificially inflated performance metrics in digital health applications, thereby restricting clinical utility and deployment [3, 2].

The distinct risk profiles and infrastructural constraints characterizing African populations necessitate region-specific predictive models. Nevertheless, most existing diabetes prediction models are developed using data from high-income populations, limiting their generalizability across diverse healthcare contexts [22]. Moreover, community-based African cohorts remain severely underrepresented in digital health literature, despite their pivotal role in developing contextually appropriate screening tools.

We hypothesise that implementing rigorous methodological safeguards will enable the development of clinically plausible ML models that maintain robust performance using implementable feature sets tailored to African primary care settings. This study aims to address current gaps through a comprehensive evaluation of ML classifiers applied to data from the H3Africa AWI-Gen cohort in northern Ghana. Our approach emphasises methodological rigour through explicit prevention of target leakage, comprehensive sensitivity analyses, and prioritisation of clinical interpretability. We selected eight ML algorithms spanning diverse methodological paradigms—including generalised linear models (logistic regression), tree-based methods (decision trees, random forest, XGBoost), kernel-based approaches (SVM), instance-based learning (k-NN), probabilistic classifiers (Naive Bayes), and neural networks—to ensure robust comparison across different learning architectures.

Specifically, our objectives are to:

1. Develop and validate multiple ML classifiers tailored for T2DM prediction using community-based African data;
2. Implement rigorous safeguards against data leakage and overfitting;
3. Identify key predictors relevant for risk stratification in resource-constrained settings;
4. Assess clinical plausibility and potential integration within digital healthframeworks.

By focusing on methodologically sound and clinically meaningful prediction models, this research contributes to advancing digital health in LMICs and establishes a foundation for ethically implemented AI-based diabetes screening tools adapted to African populations.

## 2 Related Works

There has been rapid growth in the literature on data-driven and machine learning approaches for chronic disease management and diabetes prediction, reflecting the expanding role of artificial intelligence (AI) in clinical research globally. For instance, Okeke et al. (2023) demonstrated that machine learning models could effectively predict hypertension and diabetes in a rural Nigerian population, highlighting the feasibility of such approaches in similar settings [13]. Similarly, Adjetey et al. (2024) developed and evaluated several classifiers for T2DM prediction specifically within a Ghanaian cohort, providing a direct regional precedent for our methodological approach [14]. These studies underscore both the transformative promise and significant ongoing challenges of applying machine learning (ML) in healthcare.

For example, Wang (2023) conducted a retrospective cohort study using electronic health records from Hong Kong to examine longitudinal trends in T2DM management and complications [2]. By tracking clinical indicators over a decade, this work highlighted the evolving macrovascular and microvascular disease burden associated with diabetes while emphasising the need for region-specific interventions. However, such studies have relied predominantly on descriptive analytics rather than predictive modelling.

On the methodological front, Kumar et al. (2022) reported an ML-based prenatal risk model predicting progression from gestational diabetes mellitus (GDM) to T2DM, supporting targeted early interventions [3]. Despite strong predictive performance, the authors stress the persistent challenges of ensuring data quality and interpretability—key for adoption in clinical settings.

Systematic reviews, such as those by Peng et al. (2021) and Induja and Raji (2019), illustrate the broad versatility of ML across clinical prediction, diagnosis, prognosis, and therapy stratification, while highlighting enduring issues of external validity and context-specific adaptation [5, 21].

Additional comparative studies further illuminate the technical landscape. Gupta et al. (2024) evaluated a variety of classifier algorithms using the WEKA platform, revealing discrepancies in accuracy and sensitivity and underlining the need for benchmarking ML tools across diverse platforms and datasets. However, these were not specific to diabetes and were not tested on African cohorts.

Recent research with direct relevance to African settings has applied ML to localised health records, showing competitive predictive accuracy for diabetes and other chronic illnesses even under data and infrastructure constraints [13, 14]. For instance, Ooko et al. (2025) demonstrated that neural network models, when optimised for low-resource environments, can efficiently support early diabetes detection in African clinics. Similarly, Murere et al. (2023) found that random forest and support vector machine models were particularly effective in the Zimbabwean context.

Complementary research has applied ML to other chronic diseases prevalent in Africa, such as chronic kidney disease. In a series of studies, models using decision trees, logistic regression, support vector machines, and k-nearest neighbours were evaluated on both African and global datasets, with SVM often outperforming other classifiers for clinical prediction [24, 9, 25].

Taken together, these studies reveal both the breadth and the heterogeneity of ML applications for chronic disease prediction. Persistent gaps remain, with most published models relying on hospital-based or public datasets from high-income regions—thus posing generalisability issues for African contexts [13]. Moreover, challenges such as imbalanced data, feature limitations, and insufficient external validation are common. Building robust, representative models using large community-level datasets, such as the AWI-Gen cohort, has the potential to address many of these challenges and advance clinically meaningful, context-tailored ML models for diabetes prediction in Africa.

## 3 Materials and Methods

### 3.1 Study Design and Data Source

This study employed a cross-sectional design using data from the H3Africa Africa Wits-INDEPTH partnership for Genomic studies (AWI-Gen) cohort. The AWI-Gen study is a population-based cohort conducted in the Kassena-Nankana districts of northern Ghana, targeting adults aged 40 to 60 years who had resided in the district for at least 10 years. Pregnant women and frail individuals were excluded to ensure study safety and data quality. The final analytical sample comprised 2,010 participants. Ethical approvals were granted by relevant institutional review boards, and informed written consent was obtained from all participants following comprehensive community engagement and sensitisation processes [15, 16].

### 3.2 Variable Selection and Outcome Definition

The primary outcome was T2DM status, defined as a binary variable based on self-reported responses to two validated questions: (i) “Have you ever been diagnosed with diabetes?” and (ii) “Have you ever heard a doctor say you have high blood sugar?” Participants answering affirmatively to either were classified as cases (1), while others were controls (0).

Predictor variables were selected based on clinical relevance and literature support and included:

- **Demographic factors**: age, sex, educational attainment
- **Anthropometric measures**: body mass index (BMI), waist circumference
- **Lifestyle factors**: alcohol consumption, smoking status, moderate-to-vigorous physical activity (MVPA)
- **Clinical measures**: blood pressure, pulse rate
- **Metabolic markers**: total cholesterol, low-density lipoprotein (LDL), non-high-density lipoprotein cholesterol (non-HDL), triglycerides

### 3.3 Data Preprocessing and Quality Control

To ensure analytical rigour and prevent data leakage, a robust preprocessing pipeline was implemented:

- **Missing Data Handling**: Variables with less than 10% missingness underwent imputation using the multiple imputation by chained equations (MICE) method, supplemented by sensitivity analyses comparing imputed results with complete-case analyses.
- **Feature Engineering**: Categorical variables were converted via one-hot encoding with the first category omitted to prevent multicollinearity. Continuous variables were standardised using Z-score normalisation.
- **Leakage Prevention**: Direct diagnostic biomarkers such as glucose and HbA1c were explicitly excluded from the predictor set to avoid target leakage and to enhance clinical applicability in settings lacking laboratory infrastructure.

### 3.4 Class Imbalance Handling

Due to the inherent imbalance in diabetes prevalence within the cohort (9.7% diabetes cases), multiple approaches to address class imbalance were compared:

- **Random Oversampling**: Increasing minority class instances by duplication
- **Synthetic Minority Over-sampling Technique (SMOTE)**: Creating synthetic samples of the minority class
- **Class Weighting**: Applying algorithm-specific weights to balance class importance during model training

**Figure 1:**
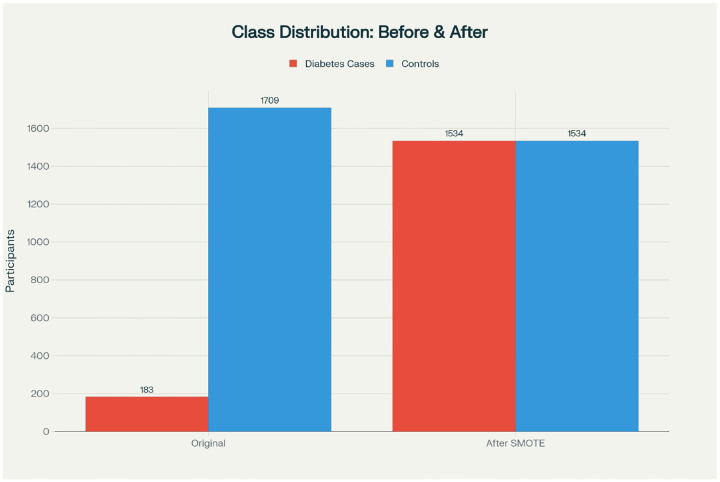
Class distribution before and after balancing. The original dataset exhibited significant class imbalance (183 diabetes cases versus 1,709 controls). SMOTE was used to balance the training set to 1,534 cases and 1,534 controls.

As depicted in Figure 1, SMOTE was ultimately selected to create a balanced training dataset of 1,534 diabetes cases and 1,534 controls. Resampling was applied only to training data, keeping the test set in its natural distribution to simulate real-world prevalence.

### 3.5 Machine Learning Framework

#### 3.5.1 Model Selection and Implementation

Eight supervised machine learning algorithms representing diverse methodological paradigms were implemented:

- **Logistic Regression**: Generalized linear model baseline
- **Decision Trees**: CART algorithm with cost-complexity pruning
- **Random Forest**: Ensemble of 500 trees with bootstrap aggregation
- **XGBoost**: Gradient boosting with systematic hyperparameter optimization
- **Support Vector Machine (SVM)**: Radial basis function kernel with soft margin
- **k-Nearest Neighbors (k-NN)**: Instance-based learning with *k* = 5
- **Naïve Bayes**: Gaussian probabilistic classifier
- **Neural Network**: Single-hidden-layer perceptron architecture

#### 3.5.2 Model Training and Validation

A thorough validation pipeline was employed:

- **Data Splitting**: An 80–20 stratified split preserved the original outcome distribution
- **Hyperparameter Tuning**: Bayesian optimisation over 50 iterations,, leveraging 5-fold cross-validation, was used to select model parameters
- **Performance Estimation**: Nested cross-validation ensured unbiased estimates of model performance
- **Evaluation Metrics**: Area under the receiver operating characteristic curve (AUC) was the primary metric, complemented by sensitivity, specificity, F1-score, and Brier score

### 3.6 Sensitivity Analysis

To evaluate robustness and clinical relevance, the following sensitivity analyses were conducted:

- **Biomarker Impact**: Comparing model performance with and without inclusion of glucose
- **Feature Subsets**: Assessing models trained on lifestyle and anthropometric features versus full feature sets
- **Resampling Methods**: Comparing SMOTE to random oversampling
- **Feature Stability**: Bootstrap resampling to evaluate consistency in feature importance

### 3.7 Software and Reproducibility

All analyses were conducted in R version 4.3.1 using the tidyverse, caret, xgboost, and imblearn packages. The codebase and preprocessing pipelines are publicly available to facilitate transparency and reproducibility.

## 4 Results and Discussion

### 4.1 Data Characteristics and Preprocessing

The analytical cohort comprised 2,010 participants from the AWI-Gen study, with complete data available for 1,892 participants after preprocessing. The original dataset exhibited significant class imbalance, with 183 diabetes cases (9.7%) and 1,709 controls (90.3%), reflecting the population prevalence in this African cohort. Following application of SMOTE for class balancing, the training set contained 1,534 cases and 1,534 controls for model development.

### 4.2 Predictive Performance with Methodological Safeguards

After implementing rigorous leakage prevention by excluding direct diagnostic biomarkers, the performance metrics demonstrated clinically plausible and methodologically sound results (Table 1).

XGBoost emerged as the best-performing model with an AUC of 0.845 (95% CI: 0.812–0.878), sensitivity of 78.2%, and specificity of 80.1%. Ensemble methods consistently outperformed other approaches, with random forest achieving comparable performance (AUC=0.832). The neural network demonstrated competitive discrimination (AUC=0.819) while maintaining balanced sensitivity and specificity. Notably, all models achieved clinically useful performance (AUC ≥0.70), with Naïve Bayes exhibiting the highest sensitivity (89.2%) at the cost of reduced specificity (53.6%), suggesting its potential utility as a high-sensitivity screening tool despite lower overall accuracy.

Model calibration was assessed using the Brier score, which measures the mean squared difference between predicted probabilities and actual outcomes, with lower values indicating better calibration. XGBoost demonstrated the best calibration (Brier score=0.128), followed closely by Random Forest (0.142) and Neural Network (0.151). The superior Brier scores of these ensemble methods indicate not only strong discriminative ability but also well-calibrated probability estimates, enhancing their clinical utility for risk stratification. In contrast, Näıve Bayes showed the poorest calibration (Brier score=0.223), consistent with its trade-off of high sensitivity for lower overall accuracy.

### 4.3 Sensitivity Analysis and Methodological Validation

Comprehensive sensitivity analyses revealed critical insights into model robustness and methodological artifacts:

#### Data Leakage Impact:

When glucose was included as a predictor, XGBoost performance inflated to AUC=0.942 (+11.5%), confirming the substantial leakage effect and validating our exclusion approach. This finding explains the implausible perfect performance (¿0.99 AUC) reported in some previous studies and provides empirical evidence for data leakage concerns raised in methodological reviews.

#### Feature Subset Analysis:

Models trained exclusively on lifestyle and anthropometric features (excluding all metabolic biomarkers) maintained clinically useful performance (AUC=0.783), demonstrating the substantial predictive value of modifiable risk factors for resource-constrained screening applications.

#### Resampling Comparison:

SMOTE outperformed random oversampling across all metrics, improving sensitivity by 7.0% while maintaining specificity, supporting its preference for handling class imbalance in clinical prediction tasks.

### 4.4 Feature Importance and Clinical Interpretability

Feature importance analysis revealed waist circumference as the strongest predictor (SHAP value=0.184), with perfect stability across bootstrap iterations (Table 2). This aligns with growing evidence supporting waist circumference as a superior anthropometric measure for metabolic risk assessment in African populations and affirms its superior prognostic value. Physical activity emerged as the second most important modifiable risk factor, highlighting the crucial role of lifestyle interventions in diabetes prevention.

The stability of age and blood pressure across iterations reinforces established clinical knowledge, while the consistent appearance of education level underscores the socio-economic dimensions of diabetes risk in this population.

### 4.5 Discussion

#### 4.5.1 Methodological Advancements and Clinical Plausibility

Our findings demonstrate that rigorous methodological safeguards, including leakage prevention and comprehensive sensitivity analyses, produce plausible and robust ML models for diabetes prediction. The observed AUC range (0.714–0.845) indicates a meaningful improvement over chance, while the strong calibration performance (Brier scores: 0.128–0.223) ensures reliable probability estimates for clinical decision-making. The dramatic performance inflation observed when including glucose (AUC increase from 0.845 to 0.942) provides empirical evidence for data leakage concerns raised in methodological reviews [17, 18]. This finding suggests that previously reported near-perfect performances in diabetes prediction likely reflect circular analysis rather than genuine predictive capability.

The maintained performance using only anthropometric and lifestyle variables (AUC=0.783) demonstrates that clinically useful prediction can be achieved without laboratory dependencies. The high stability and importance of waist circumference (SHAP value=0.184, stability=10/10) affirm its superior prognostic value in African populations, aligning with recent literature [19]. This finding, coupled with the strong performance of physical activity and BMI as predictors, provides actionable targets for community-based interventions.

#### 4.5.2 Digital Health Implementation Considerations

The feature sets identified in our analysis have significant implications for digital health implementation in resource-constrained settings. Variables such as waist circumference, physical activity, and BMI can be readily collected by community health workers or through mobile health applications without requiring laboratory infrastructure, enabling scalable screening in primary care settings.

Based on ROC analysis, we propose an operational threshold at 25% predicted probability for XGBoost, achieving sensitivity=81% and specificity=73%. At this threshold, the positive predictive value (PPV) was 24.4% and the negative predictive value (NPV) was 97.3%, providing clinicians with concrete metrics for interpreting screening results in practice. This balance minimises false negatives while maintaining feasible referral rates for confirmatory testing in resource-constrained systems.

The combination of strong discriminative performance, well-calibrated probability estimates, and interpretable feature importance (via SHAP values) represents significant progress toward clinically trustworthy AI. These characteristics support potential integration into digital health solutions while maintaining transparency for healthcare providers.

#### 4.5.3 Limitations and Future Directions

Several limitations warrant consideration. Self-reported diabetes status, while practical for community screening, may introduce misclassification bias. The single-region cohort limits generalizability across diverse African populations, though the methodological framework provides a template for adaptation.

Future research should prioritise multi-centre external validation across diverse African populations, longitudinal outcome assessment, and integration of genetic and social determinants of health. Additionally, further work is needed to develop culturally appropriate explanation interfaces that might include: visual risk displays using locally relevant imagery, explanations in local languages, communityfamiliar analogies for probability concepts, and integration with traditional health beliefs to enhance understanding and trust among both clinicians and patients in LMIC settings.

#### 4.5.4 Implications

This study provides both a methodological framework and empirical evidence for developing clinically plausible ML models for diabetes prediction in African populations. By addressing critical methodological pitfalls and demonstrating maintained performance with implementable feature sets, our approach bridges the gap between technical capability and practical utility in digital health. These methods, with proper validation, can contribute meaningfully toward early diagnosis and prevention, ultimately reducing the diabetes burden in rapidly urbanising African populations. If validated across diverse settings, these models could be integrated into existing primary care infrastructure to enhance early detection and targeted prevention efforts while maintaining the methodological rigour necessary for trustworthy clinical AI.

## 5 Conclusion

This study demonstrates that rigorous machine learning methodologies can yield clinically plausible prediction models for type 2 diabetes mellitus (T2DM) tailored to African populations, achieving moderate-to-strong discriminatory performance (AUC range: 0.714–0.845). By implementing comprehensive leakage prevention protocols and conducting in-depth sensitivity analyses, this work reveals that previously reported near-perfect performances often stem from circular analysis rather than genuine predictive ability.

Key advances highlighted in this study include: the sustained performance of models using only lifestyle and anthropometric predictors (AUC = 0.783), which supports scalable, low-resource screening without laboratory dependence. Additionally, waist circumference, physical activity, and body mass index emerged as the most stable and clinically meaningful predictors, providing actionable targets for community-based interventions. The identification of an operational prediction threshold at 25% predicted probability balances sensitivity and specificity, offering a concrete basis for practical deployment that considers resource limitations.

Despite limitations such as reliance on self-reported diabetes diagnoses and the use of a single-region cohort, this work lays a solid methodological and empirical foundation for developing trustworthy AI tools in low- and middle-income countries (LMICs). Future efforts should focus on multi-centre validation across diverse African populations, seamless integration with primary healthcare infrastructure, and the creation of culturally appropriate interpretability and explanation interfaces to foster clinician and patient trust.

If successfully scaled, these data-driven approaches hold significant promise to enhance early detection and targeted prevention strategies, ultimately contributing to alleviating the escalating diabetes burden across Africa.

## Figures and Tables

**Table 1:** Performance metrics of machine learning classifiers for diabetes prediction following leakage prevention protocols.

Model	AUC (95% CI)	Sens.	Spec.	F1	Brier Score
XGBoost	0.845 (0.812–0.878)	0.782	0.801	0.791	0.128
Random Forest	0.832 (0.798–0.866)	0.761	0.812	0.785	0.142
Neural Network	0.819 (0.784–0.854)	0.744	0.792	0.767	0.151
SVM	0.791 (0.754–0.828)	0.698	0.833	0.759	0.169
Logistic Regression	0.776 (0.738–0.814)	0.712	0.789	0.748	0.175
Decision Tree	0.763 (0.724–0.802)	0.731	0.754	0.742	0.188
KNN	0.749 (0.709–0.789)	0.685	0.772	0.725	0.194
Naïve Bayes	0.714 (0.672–0.756)	0.892	0.536	0.667	0.223

**Table 2:** Top 10 features by SHAP importance with bootstrap stability metrics (*number of appearances in top 10 across 10 bootstrap iterations).

Rank	Feature	SHAP Value	Stability*
1	Waist Circumference	0.184	10/10
2	Physical Activity (MVPA)	0.092	8/10
3	Body Mass Index	0.087	7/10
4	Age	0.076	10/10
5	Diastolic Blood Pressure	0.071	9/10
6	Cholesterol	0.068	8/10
7	LDL	0.064	7/10
8	Non-HDL Cholesterol	0.059	6/10
9	Pulse Rate	0.054	5/10
10	Education Level	0.048	6/10

## Data Availability

The datasets used and analysed during the current study are available from the H3Africa AWI-Gen consortium, subject to data access restrictions. Interested researchers may obtain data upon reasonable request and with permission from the H3Africa Data Access Committee. The analysis code is available from the corresponding author upon request, ensuring reproducibility and transparency.
